# Two novel mutations in LSS gene associated with hypotrichosis simplex in a Chinese family

**DOI:** 10.1111/jocd.16530

**Published:** 2024-08-16

**Authors:** Linfang Yang, Shengjie Li, Mengmeng Yao, Shibin Jiang, Fang Cheng, Xinmiao Jia

**Affiliations:** ^1^ Departments of Dermatology Xingtai People's Hospital Xingtai Hebei China; ^2^ Biomedical Engineering Facility of National Infrastructures for Translational Medicine, Peking Union Medical College Hospital Beijing China; ^3^ Center for bioinformatics, National Infrastructures for Translational Medicine, Peking Union Medical College Hospital Beijing China

Hypotrichosis simplex is a rare autosomal recessive abnormality characterized by hair loss disorder, typically manifesting in infancy or early childhood causing significant psychological impact on patients. Research suggests a link to mutations in the LSS gene encoding lanosterol synthase, an enzyme that converts (S)‐2,3‐oxidosqualene to lanosterol.^1^, ^2^, ^3^, ^4^ Here, we report a pediatric case of hypotrichosis simplex in a Chinese family. Using whole‐exome sequencing (WES), we identified two novel mutations in the proband's LSS gene, confirmed by Sanger sequencing. Protein modeling and Enzyme‐Linked Immunosorbent Assay (ELISA) were conducted to assess the mutations' impact on LSS protein structure and expression.

The family included one patient and three unaffected individuals (Figure 1A and 1B). The proband, a 3‐year‐old boy, exhibited sparse hair since birth, which persisted through early childhood. He comes from a non‐consanguine family with no history of baldness or clinical symptoms among parents and a 9‐year‐old brother, all of whom have normal intelligence. Apart from sparse hair, the proband did not show abnormalities in eyebrows, nails, teeth, or vision.

**FIGURE 1:**
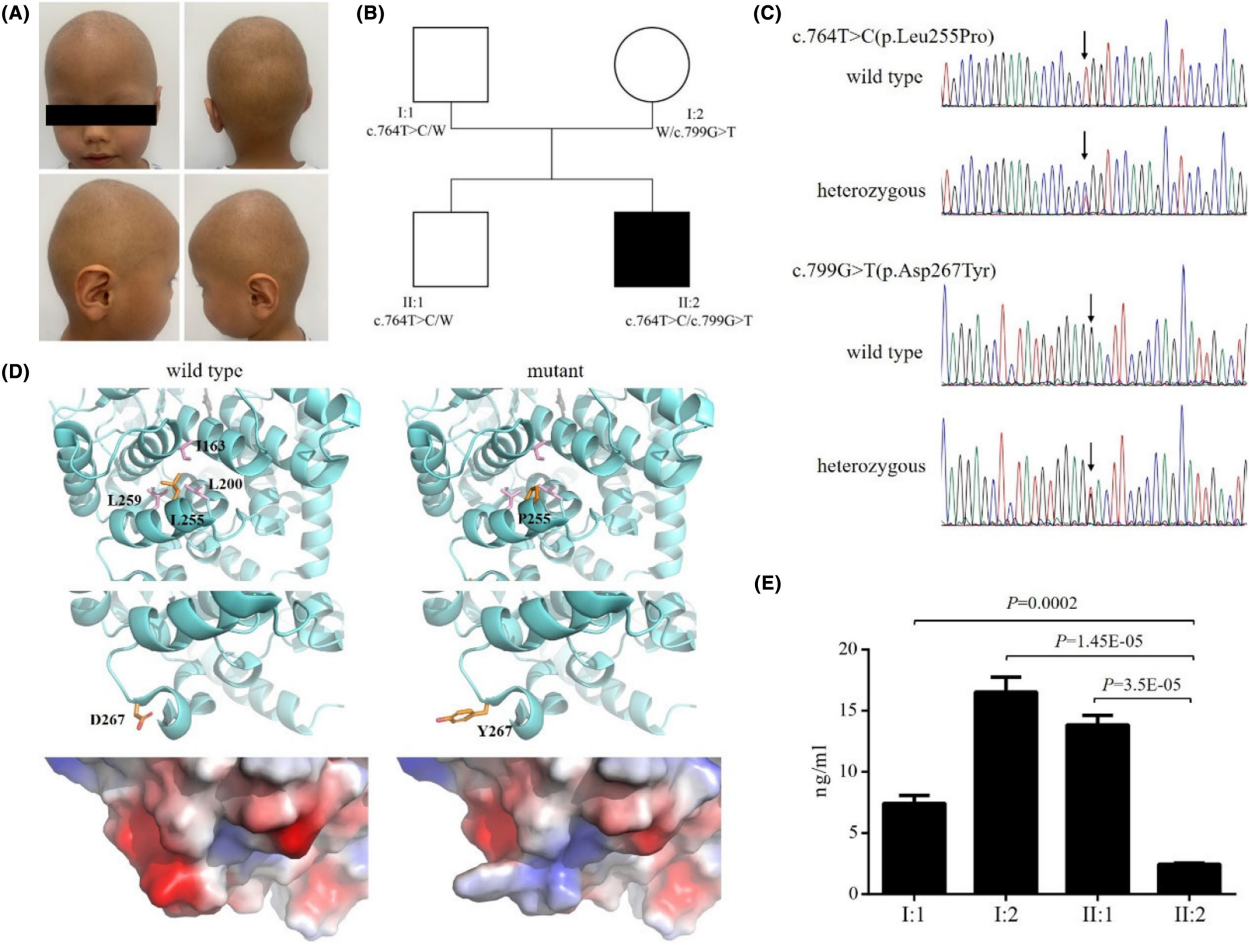
(A) Clinical pictures of the patient. (B) This family composed of one patient and three unaffected individuals. Filled square indicate the patient, empty square and circles indicate the normal individuals, the alleles of each individual are indicated below, and “W” indicates wild‐type allele. (C) Partial sequences showing the LSS c.764 T > C(p.Leu255Pro) and c.799G > T(p.Asp267Tyr) variations. Arrows point to the wild‐type sequence (up) and the mutated sequence (down). (D) Structural changes and decreased enzymatic activities of the LSS variants. (E) The expression of protein LSS in four samples detected using ELISA.

Peripheral blood samples from four individuals were collected for DNA extraction and WES. Further analysis identified two novel heterozygous mutations of c.764 T > C(p.Leu255Pro) and c.799G > T(p.Asp267Tyr) in the LSS gene in the proband, which were inherited from his father (c.764 T > C, p.Leu255Pro) and mother (c.799G > T, p.Asp267Tyr), respectively (Figure 1B). The brother of the proband only possesses one heterozygous mutation of c.764 T > C(p.Leu255Pro) just like his father does, indicating that a single mutation is not sufficient to cause the disease phenotype. Sanger sequencing further confirmed these two heterozygous mutations (Figure 1C).

SIFT, Mutation Taster, and Condel were used to predict the impact of the two heterozygous mutations of c.764 T > C(p.Leu255Pro) and c.799G > T(p.Asp267Tyr) on the function of LSS, and all three software showed “Deleterious.” Protein modeling was performed for p.Leu255Pro and p.Asp267Tyr separately to assess their impacts on protein structure (Figure 1D). Homology model was generated using SwissModel based on the wild‐type structure (PDB:1W6J). Visual inspection with PyMOL identified variant‐affected regions. Missense variants L255 and D267 were located in the domain 2 of LSS protein, with L255 on the amino terminal of α‐helix 8 and D267 on the loop between α‐helix 8 and α‐helix 9. L255 stabilizes α‐helices via hydrophobic interactions, and proline replacement could disrupt this. Also, the proline residue acts as an α‐helix disruptor, and the L255P point mutant may affect the stability of α‐helix 8. D267 occupies a surface area near the membrane binding site; the mutant tyrosine is larger and neutral, differing from the wild‐type aspartate. These changes may abnormally affect LSS function.

Using ELISA, we observed a significant reduction in LSS protein expression in the proband compared to the other three family members (Figure 1E). This indicated that the coexistence of these two mutations does have an impact on the function of LSS, contributing to the disease phenotype.

This study reports new mutations in the LSS gene, providing valuable insights into the association between hypotrichosis simplex and the LSS gene, contributing to the understanding of the genetic basis of hypotrichosis simplex.

## FUNDING INFORMATION

This work was supported by the medical science research project plan in Hebei province, China (20240923). The funders had no role in the study design, data collection and analysis, decision to publish, or preparation of the manuscript.

## CONFLICT OF INTEREST STATEMENT

None declared.

## ETHICS STATEMENT

The study was approved by the ethics committee of Affiliated Xingtai People's Hospital of Hebei Medical University (2024[020]). Written informed consent was obtained from the proband's parents.

## Data Availability

Not applicable.
